# CCL3 contributes to secondary damage after spinal cord injury

**DOI:** 10.1186/s12974-020-02037-3

**Published:** 2020-11-27

**Authors:** Nicolas Pelisch, Jose Rosas Almanza, Kyle E. Stehlik, Brandy V. Aperi, Antje Kroner

**Affiliations:** 1 Department of Neurosurgery, Medical College of Wisconsin, Milwaukee, WI 53226 USA; 2 Clement J. Zablocki Veterans Affairs Medical Center, Milwaukee, WI 53295 USA; 3 Department of Microbiology and Immunology, Medical College of Wisconsin, Milwaukee, WI 53226 USA

**Keywords:** CCL3, Spinal cord injury, Inflammation, Secondary damage

## Abstract

**Background:**

Secondary damage after spinal cord injury (SCI) is characterized by a cascade of events including hemorrhage, apoptosis, oxidative stress, and inflammation which increase the lesion size which can influence the functional impairment. Thus, identifying specific mechanisms attributed to secondary injury is critical in minimizing tissue damage and improving neurological outcome. In this work, we are investigating the role of CCL3 (macrophage inflammatory protein 1-α, MIP-1α), a chemokine involved in the recruitment of inflammatory cells, which plays an important role in inflammatory conditions of the central and peripheral nervous system.

**Methods:**

A mouse model of lower thoracic (T11) spinal cord contusion injury was used. We assessed expression levels of CCL3 and its receptors on the mRNA and protein level and analyzed changes in locomotor recovery and the inflammatory response in the injured spinal cord of wild-type and *CCL3*^*−/−*^ mice.

**Results:**

The expression of CCL3 and its receptors was increased after thoracic contusion SCI in mice. We then examined the role of CCL3 after SCI and its direct influence on the inflammatory response, locomotor recovery and lesion size using *CCL3*^*−/−*^ mice. *CCL3*^*−/−*^ mice showed mild but significant improvement of locomotor recovery, a smaller lesion size and reduced neuronal damage compared to wild-type controls. In addition, neutrophil numbers as well as the pro-inflammatory cytokines and chemokines, known to play a deleterious role after SCI, were markedly reduced in *the absence of CCL3*.

**Conclusion:**

We have identified CCL3 as a potential target to modulate the inflammatory response and secondary damage after SCI. Collectively, this study shows that CCL3 contributes to progressive tissue damage and functional impairment during secondary injury after SCI.

**Supplementary Information:**

The online version contains supplementary material available at 10.1186/s12974-020-02037-3.

## Background

Spinal cord injury (SCI) is a debilitating neurological condition with an enormous impact on the health and quality of life of affected individuals. Despite intensive research efforts and clinical improvements in the past years, there is currently no cure for SCI, which thus continues to be a significant debilitating neurological condition [1]. While the inflammatory response to the initial physical injury is essential for the clearance of cellular debris and tissue remodeling, excessive and unresolved inflammation after SCI can contribute to the secondary tissue damage and thus enhance functional impairment [2–4]. The cellular inflammatory environment after SCI is characterized by the activation of microglia, which have a mainly neuroprotective effect after SCI [5], cytokine production, the activation of astrocytes and infiltration of neutrophils, macrophages and lymphocytes all capable of contributing to the progression of secondary injury [6–9]. These immune cells release a variety of cytotoxic factors, including reactive oxygen species, pro-inflammatory cytokines, and chemokines [10, 11] such as CCL2, CXCL1, CXCL2, and CCL20. These chemokines have been reported to play a complex role in the activation and attraction of immune cells after SCI [12, 13].

Similarly, CCL3, also known as macrophage inflammatory protein α (MIP-1α), is a pro-inflammatory chemokine which plays an important role in CNS inflammation. As a member of the C-C-subfamily of low molecular weight chemokines, CCL3 is transcriptionally regulated during inflammation [14] and is detectable in most mature hematopoietic cells, such as monocytes, macrophages [15], and neutrophils [16], as well as in microglia and astrocytes [17, 18]. CCL3 expression is induced by pro-inflammatory stimulators like LPS, TNF and IL-1β [18–20], and neuronal injury [21]. CCL3 can enhance the production of other pro-inflammatory cytokines via the G-protein coupled receptors CCR1, CCR4, and CCR5 [14]. There is considerable evidence that CCL3 impacts CNS inflammation through regulation of macrophages and astrocytes. For instance, it is highly expressed in lesions of multiple sclerosis (MS) [22–24] and similarly in an animal model of MS, experimental allergic encephalomyelitis (EAE), where the onset of the disease coincides with the mRNA expression of CCL3 [25]. The absence of both CCL2 and CCL3 results in amelioration of gray matter demyelination after cuprizone treatment [26]. In the peripheral nervous system, CCL3 is upregulated in autoimmune neuritis [27] and after nerve injury [28, 29]. CCL3 has been also shown to be upregulated after SCI in both mice and rats [30–35]. Although it has also been implicated in neuropathic pain following both peripheral nerve injury [36] and SCI [37], the role of CCL3 after SCI and its direct influence on inflammation, injury progression and functional recovery have not yet been described.

Therefore, the aims of this study are to examine the role played by CCL3 in inflammation, injury progression, and functional outcome in a mouse model of moderate contusion injury to the lower thoracic spinal cord. The role of CCL3 was investigated in global *CCL3*^*−/−*^ mice on a C57BL/6J background. This strain is viable and fertile without overt hematopoietic abnormalities. Reduced clearance of viruses and reduced inflammatory response has been reported [38]. Similarly, *CCL3*^−/−^ mice showed a marked attenuation of inflammatory markers as well as lung and liver edema following trauma hemorrhage [39]. *CCL3*^*−/−*^ mice were previously used in a model of traumatic brain injury where a close interaction with CCL2 expression was demonstrated [40].

Here, we demonstrate that CCL3 and its receptors are upregulated after thoracic contusion SCI and may play a role in the cellular and molecular inflammatory cascade that ensues contusive injury to the spinal cord. Our data suggest that the absence of CCL3 after SCI helps to attenuate secondary damage and may influence the long-term functional recovery.

## Materials and methods

### Spinal cord contusion injury

All procedures were approved by and performed in accordance with the Institutional Care and Use Committees of the Clement J Zablocki VA Medical Center and the Medical College of Wisconsin. Young adult 8- to 10-week-old female wild-type C57BL/6J and *CCL3*^−/−^ mice (B6.129P2-*Ccl3*^*tm1Unc*^/J, 18–22 g) were obtained from The Jackson Laboratory, bred in our facility and used for all experiments. *CCL3*^−/−^ mice were genotyped following the genotyping protocol provided by The Jackson Laboratory. For spinal cord injury, mice were deeply anesthetized with isoflurane (4% induction, 2.5% maintenance), and a moderate contusion was induced at the T11 thoracic level using the Infinite Horizon Impactor device (Precision Systems and Instrumentation, LLC, Lexington, KY) with a contusion force of 50 or 40 kdyn, to achieve different levels of severity. Control animals were subjected to a laminectomy without contusion. Animals received subcutaneous carprofen (5 mg/kg) twice daily for 3 days post-injury. Bladders were expressed twice daily until micturition reflexes returned.

The overall study was organized in two separate cohorts of mice for behavioral assessment, as well as wild-type animals for the detection of CCL3 expression on the mRNA and protein level. In addition, mice were used for assessment of neutrophil infiltration and expression levels of cytokines and chemokines at different time points after SCI (see below).

### Locomotor assessment

The Basso mouse scale (BMS) open-field locomotor test (score range of 0–9) [41] was used to assess functional recovery during a period of 28 to 42 days. Mice (*n* = 10–15 wild-type, 9–13 *CCL3*^−/−^ mice per group) were assessed for locomotor function at day 1, 3, 7, 10, and 14 post-injury and weekly thereafter by two individuals blinded to experimental conditions and trained in BMS analysis by Dr. Basso’s laboratory at Ohio State University. Consensus scores for each animal were averaged at each time point for a maximum of 9 points for the BMS score and 11 points for the subscore, which assesses finer aspects of locomotion. This locomotor assessment was performed in three separate cohorts of mice.

### QPCR

For tissue collection, female wild-type mice were euthanized with an overdose of phenytoin/pentobarbital (120 mg/kg) without surgical intervention, after laminectomy only (at 6 h and 1, 3, and 7 days post-laminectomy) and at 1 and 6 h or 1, 3, 7, 14, and 28 days after SCI for mRNA analysis (*n* = 4–7 wt mice per time point). After transcardial perfusion with ice cold, fresh 1× phosphate-buffered saline (PBS), a 4-mm piece of spinal cord centering on the lesion was dissected out and immediately snap frozen. RNA was extracted using the RNeasy Lipid Tissue Mini Kit (Qiagen, Hilden, Germany) according to the manufacturer’s instructions, followed by RNA quantification and characterization of purity using Nanodrop (Thermo Scientific) and reverse transcription of 1 μg of RNA using QuantiTect Reverse Transcription Kit (Qiagen). Q-PCR was performed in duplicates using a PCR thermal cycler (LightCycler® 480 System, Roche) with specific primers (for sequences see Table 1) using LightCycler® 480 Mastermix (Roche). Gene expression levels were analyzed using the ΔΔct method normalized to peptidylprolyl isomerase A (PPIA) as a housekeeping gene and laminectomy as a baseline. Tissue for the comparison of inflammatory and apoptotic markers at days 1, 3, 10, and 14 after SCI was obtained and treated in the same way (*n* = 6 wild-type and 5 *CCL3*^*−/−*^ mice per group and time point).

**Table 1:** Primer sequences used for Q-PCR

Primer name	Sequence
Arginase-1 F	GTC CCT AAT GAC AGC TCC TTT C
Arginase-1 R	CCA CAC TGA CTC TTC CAT TCT T
Bax F	CAT CTT CTT CCA GAT GGT GA
Bax R	GTT TCA TCC AGG ATC GAG CAG
CCL3 F	CAG CTT ATA GGA GAT GGA GCT ATG
CCL3 R	TCA CTG ACC TGG AAC TGA ATG
CCR1 F	TCA ACT CAA CTC CAT CCA ACC
CCR1 R	CTC TGC TCC AGT TCC AGT AAA G
CCR4 F	CTT GCA CCA AGG AAG GTA TCA
CCR4 R	GGA CCA GAA CCA CAA CAG AA
CCR5 F	GCT CCA AGA GAT GAG GAA GAG
CCR5 R	GAA CAC AGA GAG CAG TCG TTA T
IL-1β F	ATG GGC AAC CAC TTA CCT ATT T
IL-1β R	GTT CTA GAG AGT GCT GCC TAA TG
IL-6 F	CTT CCA TCC AGT TGC CTT CT
IL-6 R	CTC CGA CTT GTG AAG TGG TAT AG
iNOS F	GAA CGG AGA ACG TTG GAT TTG
iNOS R	TCA GGT CAC TTT GGT AGG ATT T
PPIA F	ATG TGC CAG GGT GGT GAC TTT A
PPIA R	TGT GTT TGG TCC AGC ATT TGC C
TNF F	TTG CTC TGT GAA GGG AAT GG
TNF R	GGC TCT GAG GAG TAG ACA ATA AAG
TGF beta F	CTG AAC CAA GGA GAC GGA ATA C
TGF beta R	GGG CTG ATC CCG TTG ATT T
IL-10 F	CCC TTT GCT ATG GTG TCC TTT C
IL-10 R	AGG ATC TCC CTG GTT TCT CTT C

### Gene expression array

After harvesting spinal cord tissue of female wild-type and *CCL3*^*−/−*^ mice at days 7 and 21 after injury (*n* = 3 per group and time point), RNA was extracted and quantified as described above. This was followed by reverse transcription using the RT2 first strand kit and RT2 profiler cytokine array (PAMM-150Z) according to the manufacturer’s instructions (Qiagen). Array results were analyzed using the GeneGlobe software tools provided by the manufacturer. Only genes significantly up- or down- regulated at least 2-fold are shown.

### ELISA and western blot

Tissue was harvested as described above at days 1, 7, 14, or 28 after SCI (*n* = 7 wild-type and 4 *CCL3*^−/−^ mice per time point) and snap frozen. For ELISA, tissue was homogenized as previously described [42] and CCL3 protein concentration was quantified in duplicates using the Quantikine ELISA Kit (MMA00, R&D systems, Minneapolis, MN, USA). For Western blot, spinal cords were homogenized in ice cold RIPA Buffer (50 mM Tris, pH 8.0, 150 mM NaCl, 5 mM EDTA pH 8.0, NP-40 1%, SDS 10%) containing Complete Protease Inhibitors (Roche 11697488801) and Phosphate inhibitors (Roche 04906845001). Total protein concentration of supernatant was determined by DC Protein Assay (Bio-Rad 500-0112) based on the Lowry method. Protein (20–40 ug), was separated by the Bio-Rad Mini-PROTEAN system on Tris-HCl, 4–20% gradient gels (Bio Rad, #4561096) and transferred to Immobilon-FL PDVF membranes (Millipore #IDFL0010), incubated with primary antibodies for CCR1 (1:1000, R&D, MAB 5986, rat-anti-mouse), CCR4 (1:200, Bioss, bc-1168R, rabbit-anti-mouse), or CCR5 (1:500, Bioss, bc-2514R, rabbit-anti-mouse). Membranes were washed in TBS-T and incubated with goat anti-rat (1:10,000, Thermo Fisher #31470) or goat anti-rabbit horseradish peroxidase (HRP)-conjugated secondary antibody (1:10,000; Thermo Fisher #31460). The membranes were washed again in TBS-T and the binding of HRP-conjugated antibodies was detected by chemiluminescence (Super Signal West Femto ECL, Pierce #34094) on a Kodak imaging station. Membranes were stripped with Reblot Plus (Millipore #2504) and re-probed with monoclonal mouse anti-GAPDH (1:50,000, mouse-anti-mouse, Advanced immunology MAB 6C5) and secondary antibody goat-anti-mouse-HRP (1:10,000, Thermo Fisher #31430) to ensure equal loading of samples. The films were scanned and the changes in protein expression levels at different time points compared to laminectomy were quantified as a ratio of the chemokine receptor and GAPDH levels using Carestream Molecular Imaging software (Version 5.3.4).

### Histology and immunofluorescence

At day 1 (*n* = 3 mice/group), day 5 (*n* = 5 mice/group), or day 28 after SCI (*n* = 6 wild-type, 4 *CCL3*^*−/−*^ mice), mice were euthanized as described above, and transcardially perfused with 1× PBS, followed by perfusion fixation with cold 4% paraformaldehyde (PFA). 1-cm-long sections of the spinal cord centering on the lesion site were dissected and post-fixed in 4% PFA for 2 h, and cryoprotected in 30% sucrose solution. The cords were then embedded in OCT compound (Sakura Finetek, Japan) and serial cross-sections (14 μm) were cut and picked up on glass slides (Superfrost Plus Gold; Fisher Scientific). Immunofluorescence labeling was performed as follows. Non-specific binding was blocked by 3 h incubation in 0.5% bovine serum albumin, 0.1% Triton, and 5% normal goat serum. For primary antibodies produced in mouse, 1% of fragment goat anti-mouse IgG (1:22, 115-007-003, Jackson ImmunoResearch) was added to the blocking solution. Sections were then incubated overnight at 4 °C with the following antibodies: glial fibrillary acidic protein (GFAP) for astrocytes (1:800, Z0334, rabbit anti-mouse; DAKO, CA, USA), Ly-6B2 for neutrophils (1:500, rat anti-mouse Ly-6B.2 MCS771GA, Biorad), CD11b (1:200, MCA711G, rat anti-mouse, Bio-Rad), Anti-Neurofilament SMI-32P (1:1500, 801702, mouse anti-rabbit; BioLegend, CA, USA, and anti-CSPG (1:50, C8035, monoclonal mouse). FluoroMyelin Red for myelin content (1:150; F34652 Thermo Fisher) was combined with GFAP antibody as previously described [43]. Slides were then incubated with secondary antibodies (1:800, Alexa Fluor 488 goat-anti-mouse IgG, A11029 and Alexa Fluor 647 goat anti-rabbit; A21245, both Thermo Fisher) for 1 h at room temperature.

### Image analysis and quantification

Images were captured using a Nikon Eclipse E600 equipped with a digital camera or a Leica SP8 Confocal Microscope. Analyses of histological sections were performed using ImageJ (ImageJ-win64 2.0). The epicenter of injury in all spinal cords was identified, followed by quantification of myelin loss using Fluoromyelin-stained cross-sections of the spinal cord. Areas devoid of GFAP labeling were measured at 112 μm intervals by calculating the GFAP-devoid area as a percentage of the total area of the cross section. Quantification of neutrophils at the epicenter of the lesion was done using Fiji (ImageJ-win64 2.0).

For the quantification of astrocytes, macrophages/ microglia and SMI-32 positive profiles, a region of interest covering a 290 × 290 μm area was captured laterally adjacent to the lesion epicenter with 14 *z*-stack sections (1 μm) spanning the complete axial thickness (14 μm). Areas with CSPG labeling were calculated as a percentage of the area selected. Using ImageJ, astrocytes and microglia/macrophages were manually counted on DAPI nuclei through a continuous viewing of all z stack sections rostral to caudal to confirm individual cellular staining thereby avoiding quantification of overlapping artifacts that can be prominent in SCI given the complex, branching morphologies of CNS cell types. Similarly, SMI-32+ endbulbs were quantified through continuous viewing of all *z*-stacks. All analyses were done by a blinded investigator.

### Statistical analyses

Comparisons between two datasets were analyzed by Student’s *t* test. Data with more than two variables were analyzed by two-way repeated measures ANOVA with post hoc Tukey’s or Dunnett’s analysis. Chi-square test was used for testing relationships categories (GraphPad Prism Version 8). All *p* values < 0.05 were considered statistically significant.

## Results

### Gene expression of CCL3 is upregulated after SCI

**Fig. 1.**
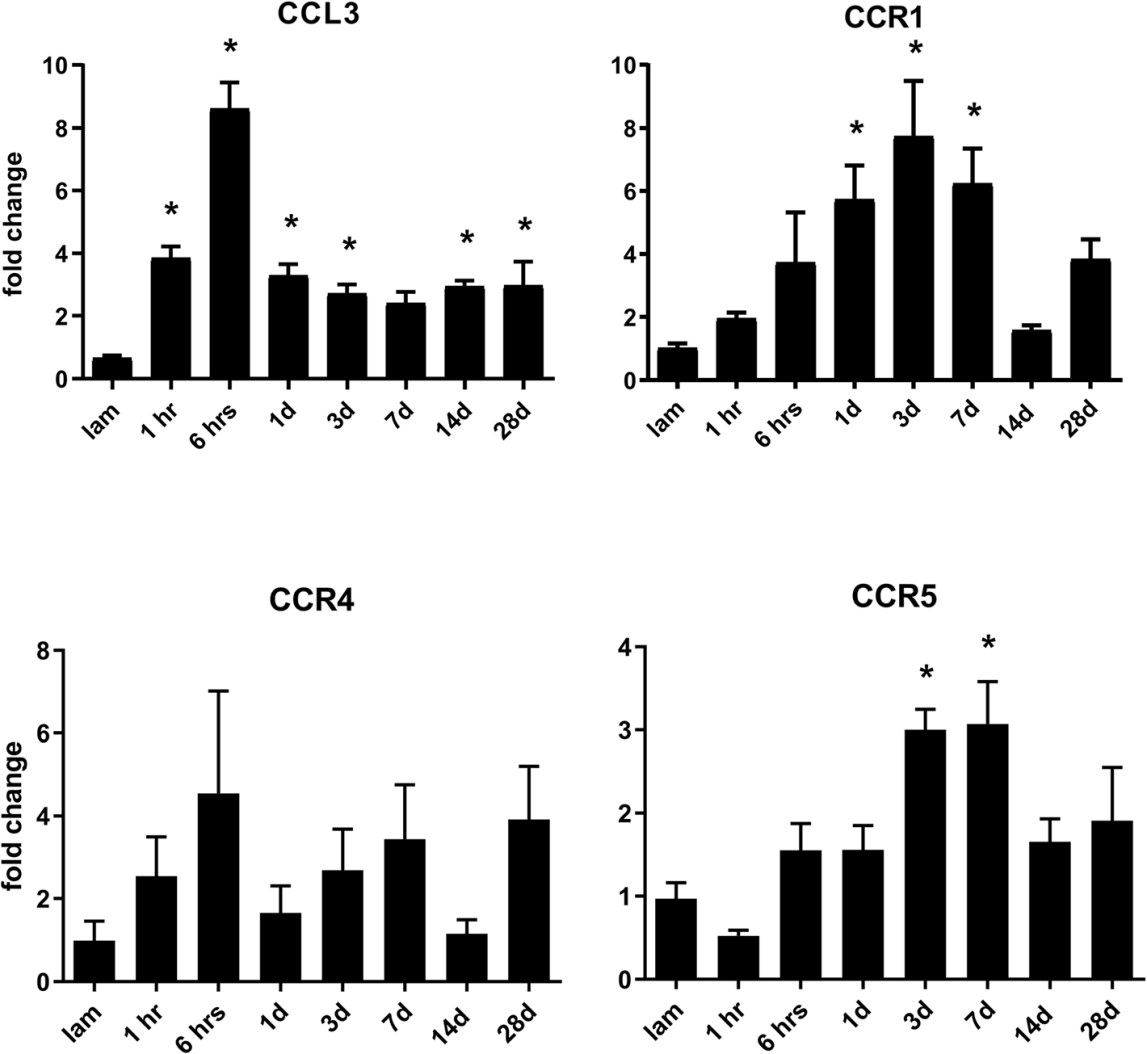
Dynamic changes in gene expression following SCI for CCL3 and its receptors. CCL3 was significantly upregulated starting at 1hr and up to 28 days post-SCI compared to uninjured (laminectomy only) mice, except on day 7 after injury. CCR1 was upregulated beginning at day 1, 3, and 7 following injury, and CCR5 was significantly upregulated at day 3 and 7 post-injury. CCR4 expression remained unchanged after SCI. Results were analyzed using the ΔΔct method by normalizing results to a housekeeping gene (PPIA) and expressed as fold changes to laminectomy control. Data are expressed as mean ± SEM. *p value < 0.05 by one-way repeated measures ANOVA followed by Tukey’s or Dunnett’s method for comparison between groups, *n* = 4–7 animals/group

We first assessed changes in the expression of CCL3 and the receptors CCR1, CCR4, and CCR5 in the injured spinal cord of female C57BL/6 mice after a moderate contusion injury (50 kdyn) at 1 and 6 h, and 1, 3, 7, 14, and 28 days following the injury. CCL3 expression increased at the lesion site as early as 1 h after injury, peaked at 6 h, and stayed significantly upregulated up to day 28 after SCI, compared to laminectomy only mice, with the exception of day 7, where only a trend to elevated levels was detected (Fig. 1). The receptors CCR1 and CCR5 were also significantly upregulated at 1, 3, and 7 days (CCR1) or 3 and 7 days (CCR5) after injury. In contrast, no changes in CCR4 expression were detected between injured and control animals at any time point (Fig. 1). In order to confirm regulation of CCL3 and its receptors after a milder contusion injury, we also assessed expression levels at the same time points using a 40 kdyn force. Comparable to the more severe injury, CCL3 expression also peaked at 6 h and stayed elevated until day 28. Both CCR1 and CCR5 showed an earlier peak at 6 h as opposed to 3 days. Interestingly, CCR4 showed a short-lived elevation at 6 h. Overall, the regulation patterns were very similar to the more severe injury (Supplemental Figure 1). We therefore proceeded to examine the protein expression in samples using 50 kdyn injury. To ensure that the expression of CCL3 and its receptors in the laminectomy controls, which served as baseline reference, did not change at different time points after laminectomy, we assessed gene expression levels at 6 h, day 1, day 3, and day 7 after laminectomy, using time points at which CCL3 expression in the injured tissue was elevated. Expression of CCL3 and its receptors in these laminectomies were normalized to naïve spinal cord samples without any surgical procedure. No changes in expression levels were detected compared to the naïve group (Supplemental Figure 2).

### Protein expression of CCL3 is upregulated after SCI

**Fig. 2.**
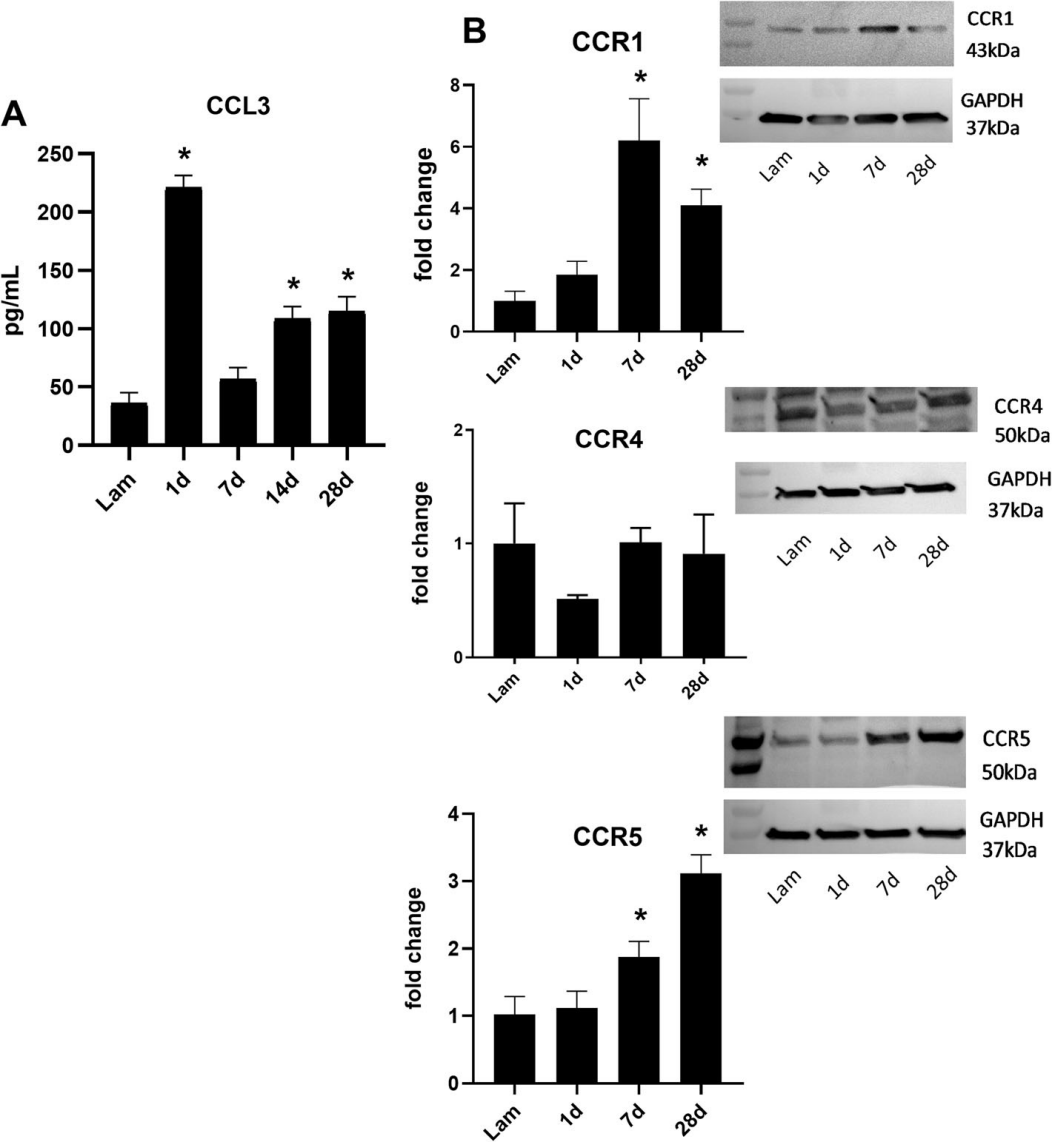
Dynamic changes in protein expression of CCL3 and its receptors following SCI. **a C**CL3 protein in spinal cord from uninjured (laminectomy only) and 1, 7, 14, 28 dpi mice was quantified using ELISA, which revealed a significant increase at day 1 following injury. By day 7, expression decreased to basal levels and was elevated again at day 14 and 28. Values are shown as mean ± SEM, *n* = 5–7 per group, *<*p* value 0.05. **b** The receptor protein expression was assessed using western blot with normalization to GAPDH. CCR1 and CCR5 were significantly upregulated at day 7 and 28 post-injury. CCR4 was not changed at any time point compared to uninjured mice. Representative images of Western blots are shown. Values are shown as mean ± SEM, *n* = 4–7 per group, * < *p* value 0.05

Next we examined the protein concentration of CCL3 and its receptors at the lesion site at several timepoints following SCI. Absolute protein levels of CCL3 were too low for detection by western blot but could be reliably detected by ELISA. Similar to the transcriptional response pattern, CCL3 concentration was significantly increased on days 1, 14, and 28 compared to laminectomy control mice (Fig. 2a). Western blot analysis of CCL3 receptors revealed a significant elevation of CCR1 and CCR5 expression on days 7 and 28 after injury compared to sham control, while CCR4 did not show any differences in protein expression through all time points (Fig. 2b).

### Absence of *CCL3* improved SCI locomotor recovery

**Fig. 3:**
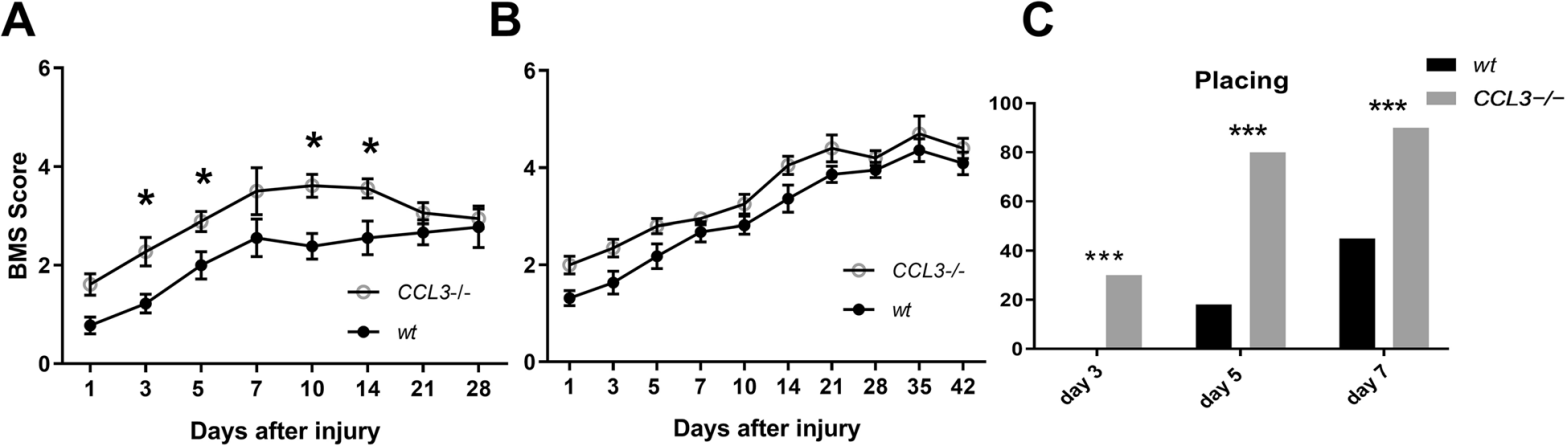
Improved locomotor recovery in *CCL3*^−/−^ mice after SCI. **a** Open-field locomotor (BMS) score shows improved recovery in *CCL3*^−/−^ compared to wild-type mice after a 50 kdyn contusive SCI at 3, 5, 10, and 14 days. Data points represent mean ± SEM (*n* = 10 wild-type, 9 *CCL3*^−/−^). *<*p* value 0.05 by two-way repeated-measures ANOVA with Tukey’s post hoc test. **b** BMS scores of an additional group of female wild-type and *CCL3*^−/−^ mice after 40 kdyn injury are not significantly different (*n* = 15 wild-type, 13 *CCL3*^−/−^). **c** For the same group as in **b**, percentage of plantar placement at days 3, 5, and 7 after injury was higher in female *CCL3*^−/−^ compared to wild-type mice (*n* = 15 wild-type, 13 *CCL3*^−/−^). Data points represent mean ± SEM. *** < *p* value 0.0001, chi-square test

The effect of CCL3 on locomotor recovery was assessed by comparing *CCL3*^−/−^ and wild-type mice. Locomotor recovery was evaluated using the BMS open field locomotor score. Before SCI, BMS scores were normal (score of 9) and did not differ between wild-type and *CCL3*^−/−^ mice. After contusive SCI (T11, 50 kdyn), *CCL3*^−/−^ mice showed improved locomotor recovery and significantly higher BMS scores on days 3, 5, 10, and 14 after SCI compared to wild-type mice (Fig. 3a). In addition, we assessed the effect of a milder contusion injury (40 kdyn) in *CCL3*^*−/−*^ and wild-type mice (Fig. 3b). A longer observation period was chosen to account for differences in locomotor recovery at later time points. In this group, BMS scores were not significantly improved in *CCL3*^*−/−*^ mice. However, a mild functional improvement was detected in the absence of CCL3, as the percentage of mice capable of plantar placement was significantly higher in *CCL3*^−/−^ (Fig. 3c) mice at day 3, 5, and 7 after injury compared to wild-type controls. When BMS data from the different experiments was pooled, there was a significant difference between wild-type and *CCL3*^−/−^ mice from day 1 through 14 after SCI (not shown).

### Absence of CCL3 reduced tissue damage after SCI

**Fig. 4.**
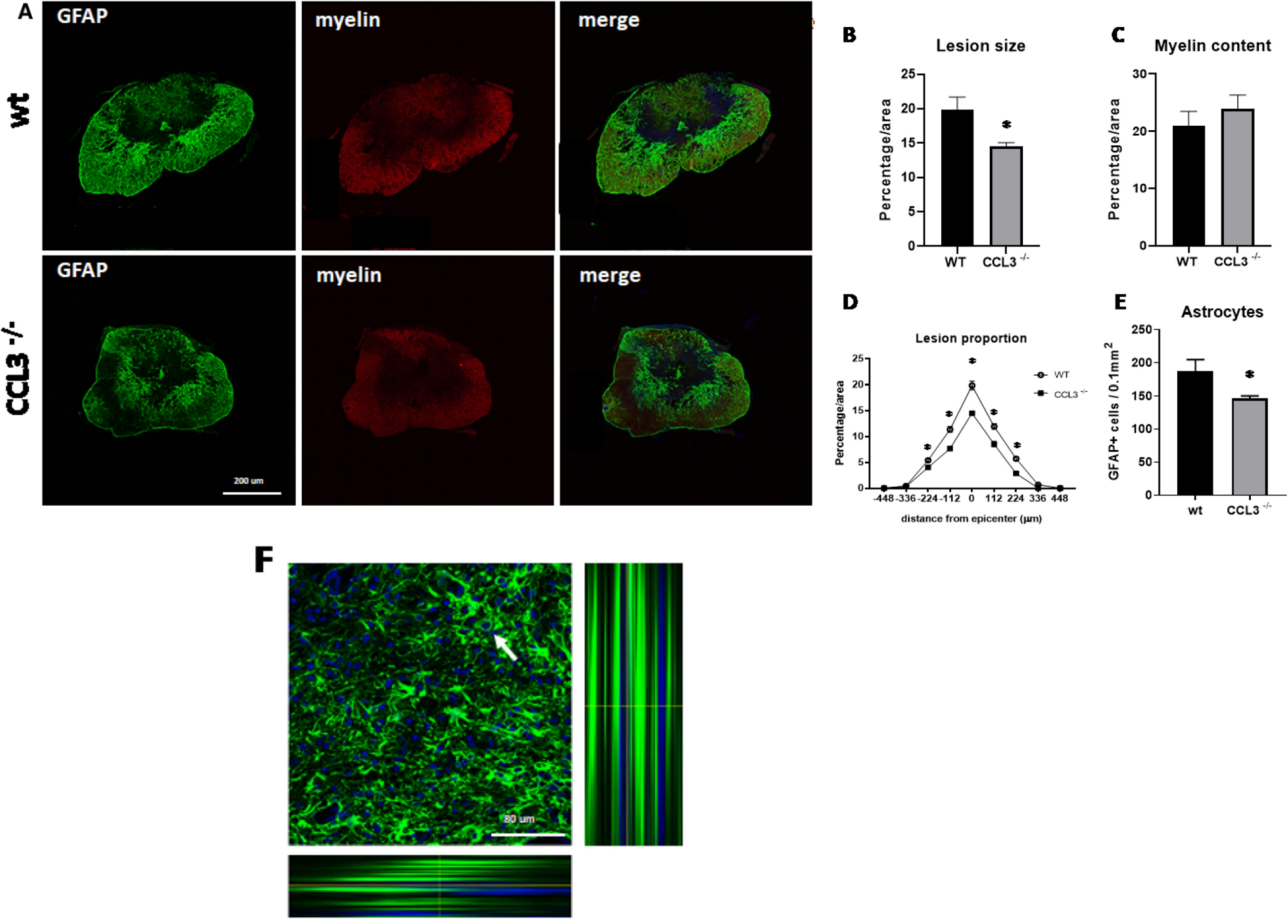
Smaller lesion sizes in *CCL3*^−/−^ mice at day 5 after SCI. **a** Representative images of GFAP immunoreactivity (green) and myelin content (red), in wild-type and *CCL3*^*–/–*^ mice at day 5 after SCI. Scale bars 200 μm. **b** Quantitative analysis revealing significantly smaller lesion size as a percentage of the total area at the epicenter in *CCL3*^−/−^ compared to wild-type mice. **c** No difference was detected in the myelin content between groups. **d** Quantitative analysis revealing significantly smaller lesion proportions in *CCL3*^−/−^ compared to wild-type mice, starting at 224 μm rostral and caudal from the epicenter. **e** Quantitative analysis revealing a significantly smaller number of astrocytes adjacent to the epicenter of the lesion in *CCL3*^−/−^ compared to wild-type mice. **f** Representative confocal image with orthogonal view of GFAP immunoreactivity (green) and DAPI (blue). Scale bars 80 μm. *n* = 5/group. Data points represent mean ± SEM. * < *p* value 0.05

Next, we analyzed different markers of cell and tissue damage and quantified lesion sizes in the injured spinal cords of *CCL3*^−/−^ and wild-type mice at different time points after injury. Immunofluorescent staining for Glial fibrillary acidic protein (GFAP) and FluoroMyelin was used to examine the lesion size as well as the amount of preserved myelin in the injured spinal cords. At day 5 after injury, quantification of areas devoid of GFAP immunoreactivity revealed significantly smaller lesion areas in *CCL3*^−/−^ compared to wild-type mice (Fig. 4a). Furthermore, quantitative analysis of the lesion proportions demonstrated a significant reduction of the lesion size in *CCL3*^−/−^ mice at the epicenter and at 112 and 224 μm rostral and caudal from the epicenter (Fig. 4b, d). The myelin content was not significantly different between *CCL3*^−/−^ mice and wild-type mice (Fig. 4c). Interestingly, *CCL3*^−/−^ mice showed significantly fewer GFAP+ astrocytes in the tissue adjacent to the lesion core (Fig. 4e, f).

**Fig. 5:**
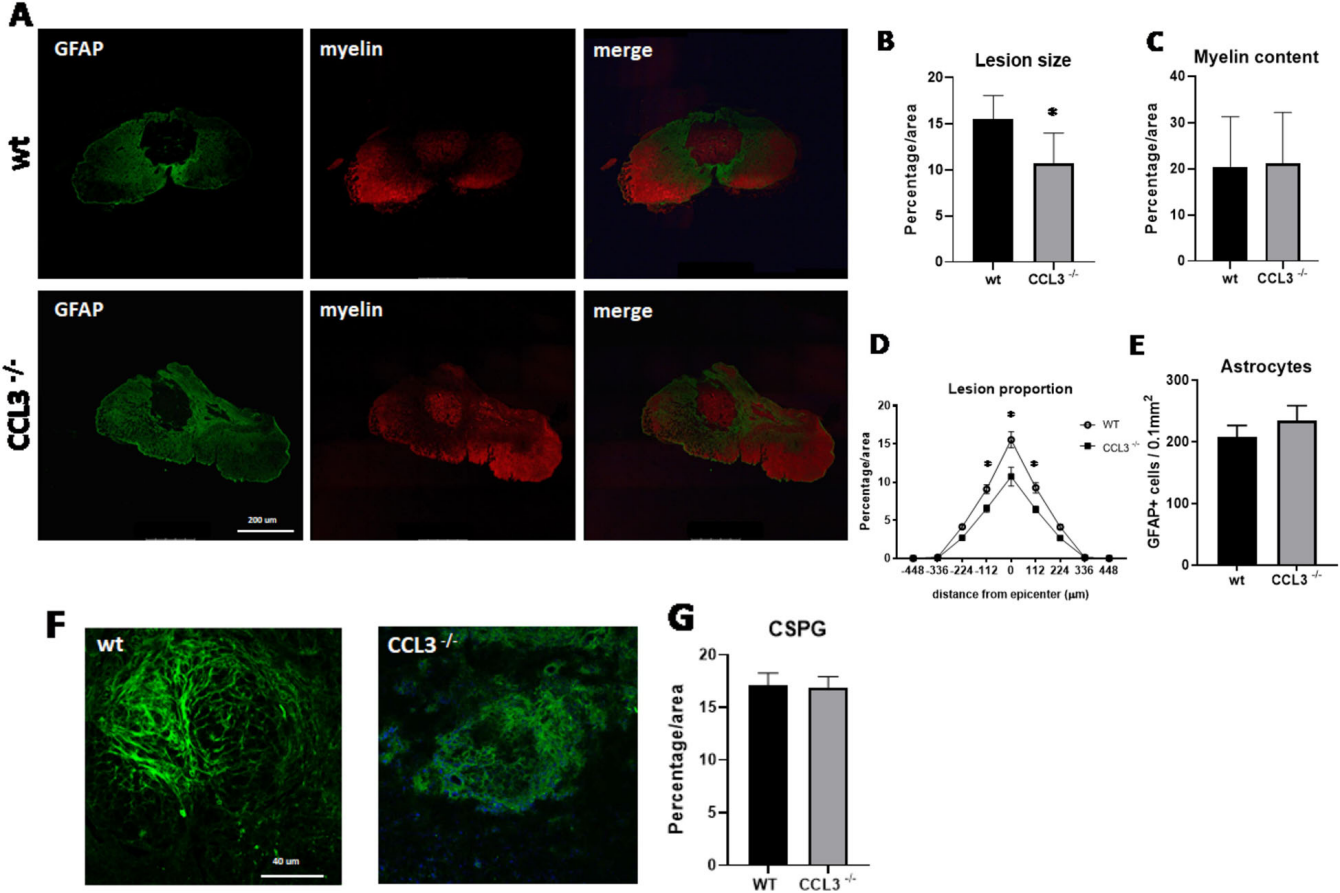
Smaller lesion sizes in *CCL3*^−/−^ mice at day 28 after SCI. **a R**epresentative images of GFAP immunoreactivity (green) and myelin content (red), in laminectomy controls, wild-type, and *CCL3*^*–/–*^ mice at day 28 after SCI. Scale bars 200 μm. **b** Quantitative analysis revealing significantly smaller lesion sizes at the epicenter in *CCL3*^−/−^ compared to wild-type mice. **c** No difference was detected in the myelin content at the lesion epicenter between groups. **d** Quantitative analysis revealing significantly smaller lesion proportions in *CCL3*^−/−^ compared to wild-type mice, starting at 112 μm rostral and caudal from the epicenter. **e** Quantitative analysis revealing no difference between groups in the number of astrocytes adjacent to the lesion epicenter. **f** Representative images of CSPG immunoreactivity (green) and DAPI (blue). Scale bars 40 μm. **g** Quantitative analysis shows no difference between groups in the amount of CSPG deposits at day 28 post-SCI. *n* = 6 wild-type, 5 *CCL3*^−/−^. Data points represent mean ± SEM. * < *p* value 0.05

Similarly, at day 28 after injury, a significantly smaller lesion area was detected in *CCL3*^−/−^ mice compared to wild-type mice (Fig. 5a). Quantitative analysis of the lesion proportions demonstrated a significant reduction of the lesion size in *CCL3*^−/−^ mice at the epicenter and at 112 μm rostral and caudal from the epicenter (Fig. 5b, d). No differences in myelin content, as shown with FluoroMyelin Red staining, were detected between groups (Fig. 5c). Unlike day 5 after injury, the number of GFAP+ astrocytes adjacent to the lesion core did not differ between *CCL3*^−/−^ and wild-type mice (Fig. 5e). At the same time point, the area stained for CSPG, a component of the glial scar, showed no significant difference between the groups (Fig. 5f, g). The difference of lesion sizes between wild-type and *CCL3*^−/−^ mice both at day 5 and day 28 after injury is suggestive of an early and sustained CCL3 dependent process of secondary tissue damage.

**Fig. 6:**
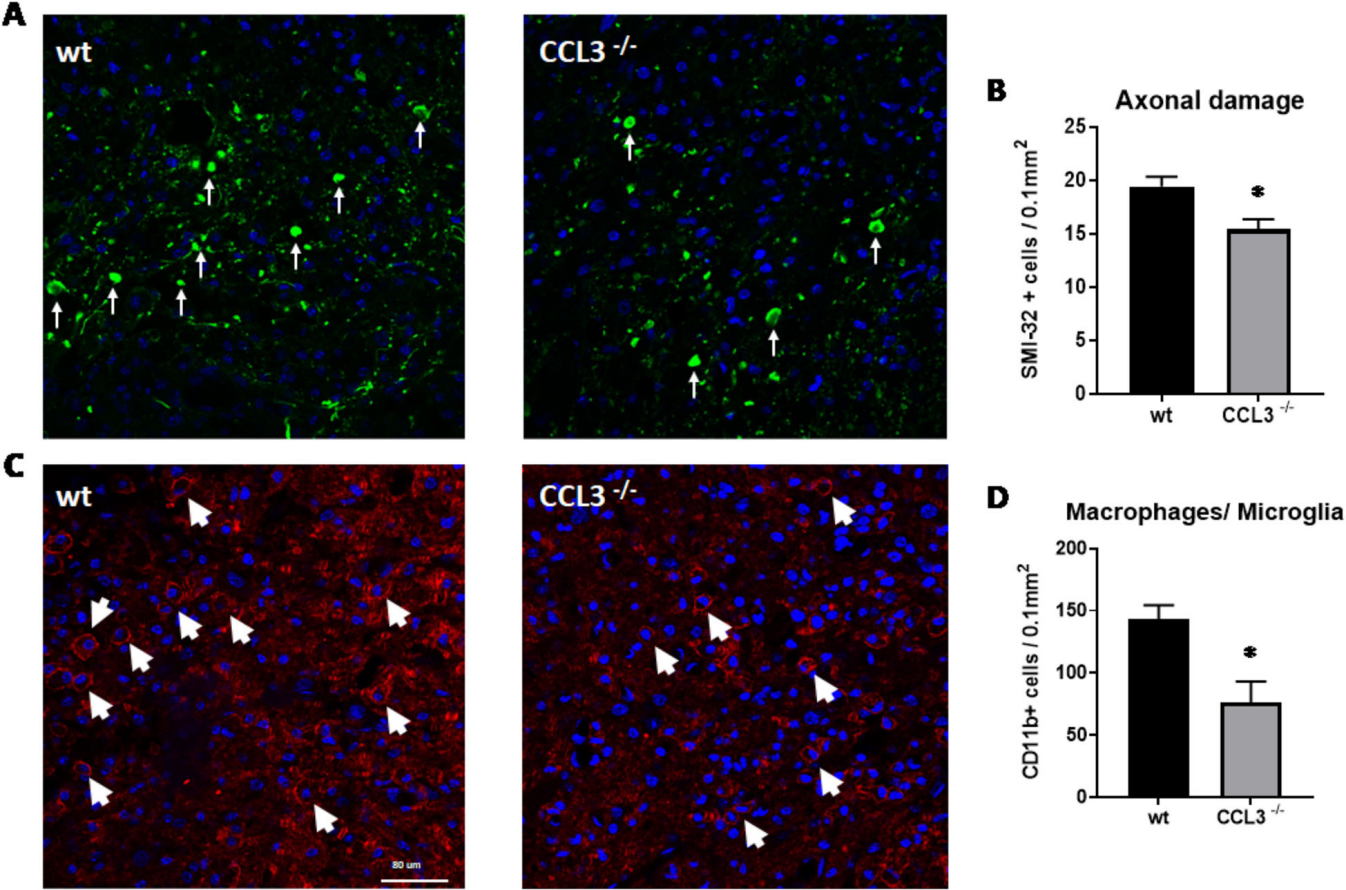
Absence of CCL3 reduces axonal damage and number of macrophages/microglia after SCI. a Representative images of SMI-32 (green) and DAPI (blue) in wild-type and *CCL3*^*–/–*^ mice at day 5 after injury adjacent to the lesion epicenter. Arrows indicate SMI-32+ profiles. **b** Quantitative analysis revealing a reduced number of SMI-32+ cells in *CCL3*^*–/–*^ compared to wild-type mice. **c** Representative images of CD11b (red) and DAPI (blue) in wild-type and *CCL3*^*–/–*^ mice at day 5 after injury adjacent to the lesion epicenter. Arrows indicate CD11b+ staining in relation to a DAPI+ nucleus. **d** Quantitative analysis revealing a reduced number of CD11b+ cells in *CCL3*^*–/–*^ compared to wild-type mice. Scale bar = 80 μm. *n* = 5/group. * = *p* value < 0.05. Data points represent mean ± SEM

To assess additional aspects of tissue damage, we compared neuronal damage between wild-type and *CCL3*^−/−^ mice at day 5, utilizing SMI-32, a marker of axonal damage. Interestingly, the amount of SMI-32+ endbulb like profiles was reduced in *CCL3*^−/−^ mice at the level of the injury epicenter and adjacent to the lesion (Fig. 6a, b). At the same time point, we detected a significantly lower amount of CD11b+ macrophages/microglia adjacent to the lesion epicenter in the injured spinal cords of *CCL3*^−/−^ compared to wild-type mice (Fig. 6c, d). This may indicate that the absence of CCL3 generates a more favorable environment conducive to neuroprotection.

### Reduced inflammatory response in *CCL3*^−/−^mice after SCI

**Fig. 7:**
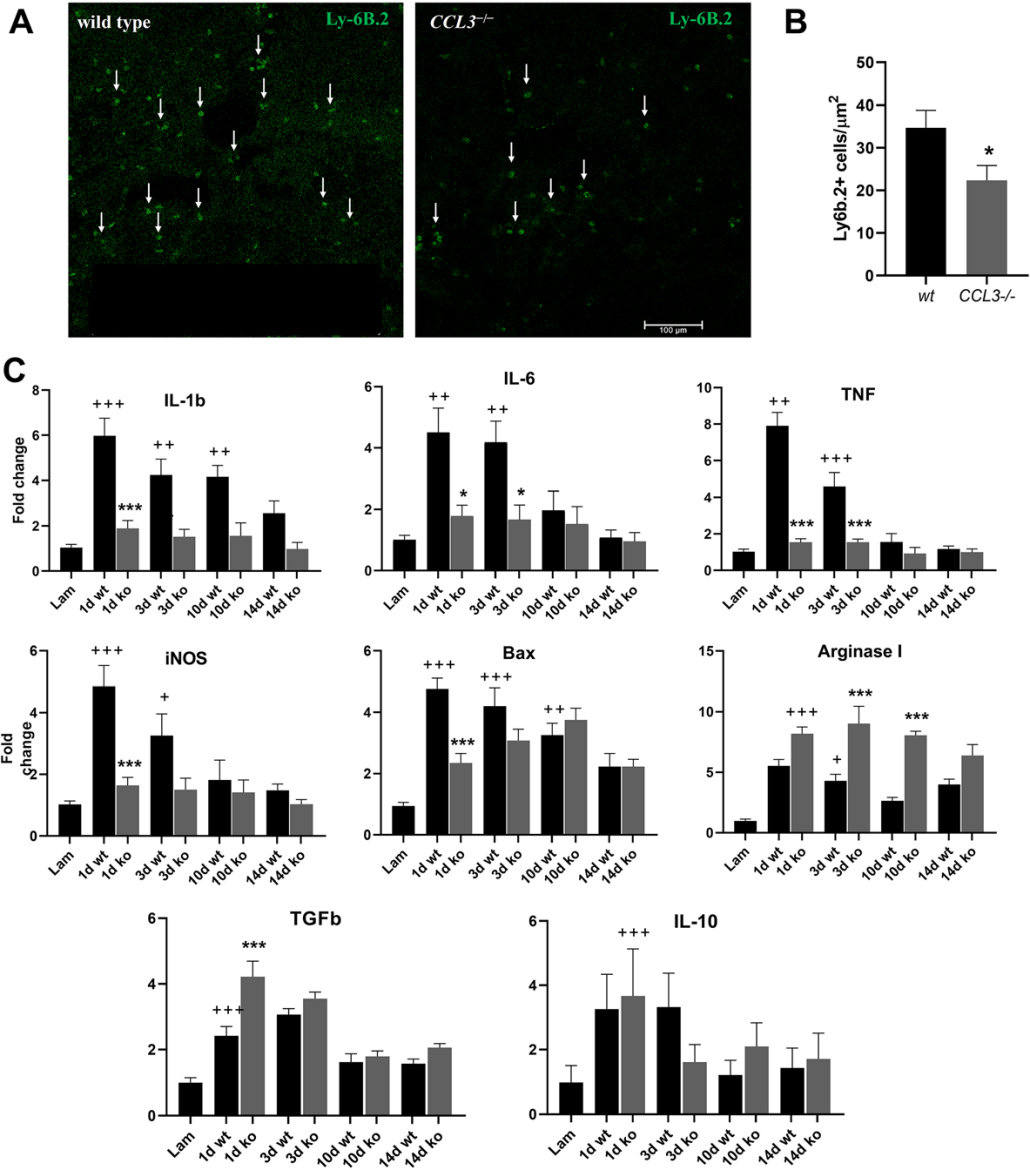
Inflammatory response in *CCL3*^−/−^ mice is reduced after SCI. **a** Representative images of Ly-6B.2-positive neutrophils in wild-type and *CCL3*^*–/–*^ SCI tissue at the lesion epicenter at day 1 after injury. Scale bar = 100 μm. Arrows indicate Ly-6B.2-positive profiles. **b** Quantitative analysis of neutrophil recruitment shows a significant reduction of neutrophils at the lesion epicenter of *CCL3*^*–/–*^ mice. *n* = 3/group. * = *p* value < 0.05. **c** Expression levels of the pro-inflammatory cytokines *il-1b*, *il-6*, *tnf*, *inos*, and the apoptotic marker *bax* were significantly increased at different time points in wild-type mice compared to laminectomy controls. *CCL3*^*–/–*^ mice, however, showed significantly lower expression levels compared to wild-type. *Arginase-1* and *TGFb*, which can indicate anti-inflammatory properties, were significantly upregulated in *CCL3*^*–/–*^ mice in relation to wild-type mice. IL-10 was upregulated compared to the laminectomy control but did not differ between genotypes. *n* = 6 wild-type, 5 *CCL3*^*–/–*^ mice. + = *p* value < 0.05, ++ = *p* value < 0.01, +++ = *p* value < 0.001 (wild-type compared to laminectomy control), * < *p* value 0.05, *** < *p* value 0.0001 (*CCL3*^*–/–*^ compared to wild-type, same day)

Subsequently, we examined the impact of the absence of CCL3 on the inflammatory response and apoptosis-related mechanisms during secondary damage. For this, Ly-6B.2+ neutrophils were quantified in a region of interest lateral to the lesion epicenter. One day after SCI, significantly less neutrophils were recruited to the lesion epicenter of *CCL3*^*−/−*^mice compared to wild-type mice (Fig. 7a, b). Also, to detect differences in cytokine expression between wild-type and *CCL3*^*−/−*^mice, Q-PCR analysis was performed on SCI tissue taken at days 1, 3, 10, and 14 after SCI. As expected*, il-1b, il-6, tnf*, and *inos* were strongly upregulated in wild-type mice at different timepoints after SCI, but the expression was markedly decreased in *CCL3*^−/−^ mice 1 and 3 days after injury, with expression levels close to the laminectomy control (Fig. 7c). *Bax*, which is involved in the apoptotic pathway and known to be upregulated in response to injury [44, 45], showed increased expression in wild-types at days 1, 3, and 10 following SCI, but was downregulated in the absence of CCL3 at days 1 and 3. *Bax* levels 10 days after SCI were not significantly different between wild-type and *CCL3*^−/−^ mice (Fig. 7c). We next sought to determine whether CCL3 contributes to the anti-inflammatory response following SCI. For this, we quantified expression levels of *type I arginase* (*Arg-I*), *TGFb*, and *IL-10*, which are found in macrophages with anti-inflammatory properties. *TGFb* was significantly upregulated in wild-type mice at days 1 and 3 after injury compared to laminectomy tissue and *CCL3*^−/−^ mice displayed significantly higher levels of *TGFb* than wild-type mice at day 1 after SCI. IL-10 was significantly upregulated in both groups on day 1 after SCI. However, IL-10 was not differentially expressed between groups. Arginase-1 was significantly elevated after injury in both wild-type and *CCL3*^−/−^ mice after SCI, with significantly higher upregulation in *CCL3*^−/−^ mice at days 3 and 10 after SCI. Overall, these results are indicative of a tissue environment with reduced pro- and increased anti-inflammatory factors.

To more broadly assess expression changes of cytokines and chemokines over time, a targeted PCR array for cytokines and chemokines was performed. We chose time points where *CCL3*^−/−^ mice demonstrated better functional properties than wild-type mice (day 7) compared to later time points, where these differences could not be seen anymore (day 21). Interestingly, at day 7 after injury, various cytokines and chemokines involved in the inflammatory response such as *il12b*, *il1a*, *tnf*, and *il17f* were downregulated in *CCL3*^−/−^ mice (for a complete list of regulated factors see Table 2). However, some anti-inflammatory regulators like *il1rn* or *bmp7* were downregulated in the absence of CCL3 as well. At day 21 after injury, only very few genes were differentially expressed between wild-type and *CCL3*^−/−^ mice, indicating a reduced difference in the cytokine and chemokine response between the different genotypes at this later time point (Table 2).

**Table 2:** Fold regulation of gene expression in *CCL3*^*−/−*^mice compared to wild-type mice at day 7 or 21 after SCI

Gene	Fold regulation	***p*** value	Function
**Day 7**			
B2m (Beta 2 microglobulin)	− 2.97	0.046	Component of MHC class I molecule [46]
Bmp7 (Bone morphogenetic protein 7)	− 3.24	0.036	Anti-inflammatory protein, neuroprotective function [47, 48]
CCl4 (CCL4)	− 5.27	0.009	CCR1 agonist, role in wound repair and inflammation [49]
Ccl11 (CCL11, eotaxin-1)	− 5.98	0.021	Eosinophil chemotactic protein [50]
CCl12 (CCL12)	− 4.39	0.035	Pro-inflammatory cytokine [51]
Cd70 (CD70)	− 3.52	0.009	Pro-inflammatory, promoting T cell responses [52]
Cx3cl1 (CX3CL1, Fractalkine)	− 5.36	0.003	Chemotactic cytokine [53]
Ifna2 (IFNα2)	− 5.3	0.003	Pro-inflammatory cytokine with role in anti-tumor immunity [54]
Il12b (IL12p40)	− 4.90	0.008	Pro-inflammatory cytokine [55]
Il16 (IL16)	− 9.67	0.049	Chemoattractant for lymphocytes and macrophages [56]
Il17f (IL17F)	− 5.24	0.001	Pro-inflammatory cytokine [57]
Il1a (IL1α)	− 2.25	0.002	Pro-inflammatory cytokine [58]
Il1rn (IL1 receptor antagonist)	− 13.93	0.015	Anti-inflammatory protein [59]
Il27 (IL27)	− 3.16	0.019	Anti-inflammatory protein [60]
Mif (Macrophage migration inhibitory factor)	− 2.54	0.005	Pro-inflammatory cytokine [61]
Osm (Oncostatin M)	− 8.96	0.041	Protein with role in inflammation, hematopoiesis [62]
Thpo (Thrombopoietin)	− 19.65	0.008	Megakaryocyte growth and development factor [63]
Tnf (TNF)	− 3.85	0.034	Pro-inflammatory cytokine [64]
**Day 21**			
Bmp6 (Bone morphogenetic protein 6)	− 2.32	0.034	Iron metabolism [65]
Ifng (IFNγ)	− 4.2	0.031	Pro-inflammatory cytokine [66]
Pf4 (Platelet factor 4/CXCL4)	− 2.76	0.008	Pro-inflammatory chemokine [67]
Thpo (Thrombopoietin)	− 2.89	0.036	Megakaryocyte growth and development factor [63]
Tnfrsf11b (Osteoprotegerin)	− 14.42	0.031	Inhibitor of osteoclast function [68]

Gene expression changes were assessed using RT2 profiler cytokine array (PAMM-150Z), *n* = 3 mice/group and time point

## Discussion

Neuroinflammation plays a critical role in secondary damage that follows traumatic SCI. In particular, pro-inflammatory cytokines and chemokines have been shown to play a critical role in exacerbating the secondary injury that follows the primary injury to the central nervous system. This study examined the effects of CCL3, a pro-inflammatory chemokine with a role in neuroinflammatory conditions, on tissue damage and locomotor recovery after contusion SCI. In female C57BL/6J wild-type mice, we detected an increase in the expression of CCL3 and the receptors CCR1 and CCR5 in the spinal cord that persisted long-term after the injury. To analyze the impact of CCL3 after SCI, using the *CCL3*^−/−^ mice, we demonstrated a mild improvement of locomotor recovery, reduced lesion sizes as well as a reduction of the inflammatory response, characterized by the neutrophil influx and expression of pro-inflammatory cytokines.

Several studies have addressed the expression of CCL3 in the injured spinal cord. For instance, in mice and rats, CCL3 is upregulated within the first day after SCI [30–33] and stays elevated for prolonged periods, with observation times of up to 42 days after injury [34, 35, 37]. A similar pattern of CCL3 regulation has been observed after traumatic brain injury in mice [40, 69]. These results are in accordance with our findings regarding the expression levels of CCL3 mRNA or protein, which were equally upregulated at early time points after SCI and stayed elevated long-term.

Similarly, we are reporting elevated levels of the receptors CCR1 and CCR5 after SCI. CCR1 mRNA expression was elevated at day 1 after injury and remained upregulated until day 7, while protein expression levels were elevated at day 7 to day 28. CCR5, whose ligands include CCL3, CCL4, CCL3L1, and CCL5, showed mRNA upregulation at days 3 and 7 after injury, while protein expression was increased by day 7 and maintained on a high level until day 28 after injury. In a rat model of SCI, CCR1 expression is also increased depending on injury severity, but shows the highest levels at later time points after SCI [34]. After mouse traumatic brain injury, in contrast, CCR1 mRNA is upregulated after injury but returns to normal levels after 7 days in cortex, striatum, and thalamus, while staying upregulated in the hippocampus for up to 5 weeks [69]. However, CCR5 mRNA stays elevated for several weeks after traumatic brain injury, while protein levels for both CCR1 and CCR5 return to baseline after 7 days [69]. The observed similarities highlight a role for CCL3 and its receptors after traumatic injuries to the CNS. The differences in expression duration could be caused by species differences and variations in tissue expression.

The third receptor CCL3 can interact with is CCR4 [70]. In our model of thoracic contusion SCI, no significant regulation of CCR4 was detected in the injured tissue at either time point, neither on the mRNA nor on the protein level. CCR4 is expressed on T regulatory (Tregs) or type 2 T-helper (Th2) cells and has previously been detected on FoxP3/GFP+ Tregs after SCI, using flow cytometry [71]. We cannot exclude that the Q-PCR on the whole injured spinal cord tissue is not sensitive enough to detect CCR4 expression levels on the individual cell level.

After demonstrating the regulation of CCL3 and its receptors CCR1 and CCR5 after SCI, we assessed the impact of CCL3 on locomotor recovery and secondary damage in *CCL3*^*−/−*^ mice compared to wild-type controls. In a first experiment, *CCL3*^*−/−*^mice initially showed significant improvement of locomotor recovery. However, at later time points, the behavioral scores of *CCL3*^*−/−*^ mice deteriorated and were no longer different from wild-type mice. BMS scores at day 1 after injury are slightly but not significantly different. We and others have previously observed a certain variability in the degree of hindlimb movement at day 1 after injury [72, 73]. In a follow-up experiment, we used a slightly milder contusion force (40 kdyn). Our objective was to investigate an injury paradigm with a slightly better recovery to detect more locomotor differences while at the same time maintaining a pronounced functional deficit. We also included a longer observation time up to day 42 following injury to determine the effect of CCL3 on recovery under these conditions. While the BMS scores were elevated using this setup, we only detected a non-significant trend to improved outcome in *CCL3*^*−/−*^ mice. A more detailed analysis of the locomotor behavior early after injury to showed that a significantly higher percentage of *CCL3*^*−/−*^ mice demonstrated plantar placement at 3, 5, and 7 days after injury.

The mild functional improvement was reflected by smaller lesion sizes in *CCL3*^*−/−*^ mice, affecting mainly the gray area, while the myelin content was not significantly different between wild-type and *CCL3*^*−/−*^mice. This effect was observed at day 5 and day 28 after injury, suggesting a sustained progression in secondary damage, which is at least partially mediated by CCL3. This is further supported by a reduction of neuronal damage, as indicated by reduced SMI-32+ profiles in *CCL3*^*−/−*^ mice compared to wild-type mice 5 days after injury.

GFAP+ astrocytes were significantly reduced adjacent to the lesion epicenter at 5 days in *CCL3*^*−/−*^mice. Twenty-eight days after injury, however, these differences between groups were not detectable anymore. This may suggest that activation or proliferation of astrocytes was delayed in *CCL3*^*−/−*^ mice after injury, but later returned to wild-type levels. Alternatively, recruitment of astrocytes may also be attenuated in a similar time frame. Similarly, detection of CSPG, a component of the glial scar, was not different between the groups at day 28. Astrocyte numbers and glial scar formation closely resembling wild-type mice at day 28 after SCI could explain an absence of functional differences at the late phase after SCI. It cannot be excluded that this effect is influenced by compensatory mechanisms in the global *CCL3*^*−/−*^ mice.

However, the detrimental effect of CCL3 and its pathway has also been demonstrated in other disease models. *CCL3*^*−/−*^ mice show reduced lesion sizes and plaque formation in atherosclerosis [74] and blockade of CCR5, using neutralizing antibodies, promotes locomotor recovery after SCI [75]. Additionally, it is important to note that CCR5 is the receptor for various chemokines besides CCL3. In addition to functional recovery after SCI, CCL3 also plays a well described role in neuropathic pain. Higher levels of CCL3 were associated with the impaired locomotor recovery and neuropathic pain in a rat SCI model [34]. After chronic constriction injury (CCI) of the sciatic nerve, CCL3, CCR1, and CCR5 mRNA and protein are upregulated in the spinal cord. Neuropathic pain could be inhibited by blockade with CCL3 neutralizing antibodies [76] or Maraviroc, a CCR5 antagonist [35]. In our model, however, no differences in thermo- or mechano-sensitivity were detected between wild-type and *CCL3*^*−/−*^ mice (data not shown). A possible explanation for this could be the differences in pain development between central and peripheral nervous tissue. Next, we investigated the mechanism by which CCL3 deficiency promotes functional recovery and reduced tissue damage. CCL3 is an inflammatory chemokine, which can induce the production of pro-inflammatory cytokines and the chemotactic mobilization of immune cells into inflammatory tissues [77].

In our SCI model, the absence of CCL3 strongly reduced the early influx of neutrophils into the injured tissue, which might contribute to the functional improvement and reduced tissue damage. This result is further corroborated by a study describing that CCL3 mediates neutrophil migration in an ovalbumin-challenged immune inflammation model via release of TNF [78]. In the spinal cord, like in many other injured tissues [79], neutrophils are the first cell type to infiltrate into the tissue, peaking 1 day after injury followed by a quick reduction in numbers [80]. Several studies have identified neutrophils as a promising target after SCI [7, 81, 82]. However, in other studies, neutrophil depletion has led to impaired recovery and increased tissue damage [83]. CCL3 and its receptors CCR1 and CCR5 are important mediators of intravascular adherence and transmigration of neutrophils [84, 85]. Absence or inhibition of CCL3 also leads to reduced neutrophil invasion and tissue protective effects in atherosclerosis [74], lung ischemia-reperfusion injury [86] and focal cerebral ischemia [87]. Similarly, CCR5 blockade results in reduced neutrophil recruitment [75] after SCI. The influence of CCL3 on immune cell infiltration is not limited to neutrophils.

The absence of CCL3 also mediated a reduced response of macrophages and microglia, as indicated by a reduction of CD11b+ cells next to the lesion site in *CCL3*^*−/−*^ compared to wild-type mice at day 5 after SCI. Overall, the CCL3 has an important role in modulating the immune response in injury and disease. Lysophosphatidylcholine (LPC) injection into the uninjured or hemisected spinal cord results in rapid and transient upregulation of CCL3 and recruitment of T cells, neutrophils and monocytes [29, 88]. An overall reduction of infiltrating immune cells in the absence of CCL3 could contribute to the tissue immune response in the injured spinal cord of *CCL3*^*−/−*^mice, which was characterized by decreased expression levels of the pro-inflammatory cytokines *il-1β*, *il-6*, and *tnf* at early time points after injury. TNF was previously indicated as a regulator of neutrophil migration under control of CCL3 [78]. Importantly, *tnf* is not only a potent pro-inflammatory cytokine in the CNS, which is known to be regulated following acute and chronic inflammatory insults [89], but also it has been described as a potent inducer of apoptosis, contributing to the pathophysiology of many neurological disorders [90]. Consistent with this, *bax*, which is involved in the apoptotic pathway, was upregulated in wild-type mice after SCI, but levels were reduced in the absence of *CCL3*. Thus, our results show that a reduction of *tnf* gene expression in the absence of CCL3 may decrease apoptosis after SCI. Further investigation is needed to determine the role of CCL3 in the apoptosis pathway during secondary damage after SCI.

Similarly, *iNOS*, as a direct marker of a pro-inflammatory reaction, was significantly decreased in the absence of CCL3. In contrast, *arginase-1 and TGFb*, which are often described as indicators for anti-inflammatory phenotypes [91], were upregulated in *CCL3*^*−/−*^mice. IL-10, which also has anti-inflammatory properties, was upregulated in both wild-type and *CCL3*^*−/−*^mice compared to the laminectomy control, but did not differ between the genotypes. It is important to consider that arginase-1 and IL-10 can also be present and upregulated in inflamed tissue, and that an upregulation of one of these factors is not necessarily an indicator for reduced inflammation. However, the combined reduction of pro- and increase of anti-inflammatory responses is suggestive of a less inflamed tissue environment and is in accordance with findings in CCR5 blockade after SCI, where anti-inflammatory markers were increased as well [75].

These findings are further supported by results form a targeted gene array for cytokines and chemokines. We compared expression levels in the spinal cord of wild-type and *CCL3*^*−/−*^mice at 7 and 21 days after injury. At 7 days after injury, *CCL3*^*−/−*^mice downregulated 19 genes and did not upregulate any genes out of 84 genes screened. The majority of downregulated genes were pro-inflammatory cytokines and chemokines. This included chemotactic proteins, like fraktalkine [53] and *il16*, which is elevated in the plasma of SCI patients [92] and in rat SCI tissue, where it likely plays a role in immune activation and cell recruitment [93]. Pro-inflammatory cytokines with reduced expression levels in *CCL3*^*−/−*^ mice included *tnf*, which negatively impacts functional recovery after SCI [73], and *mif*, which is also elevated in the plasma of SCI patients [92], has neurotoxic properties [94] and is detrimental after SCI in mice [95]. Interestingly, the anti-inflammatory factors *il1ra* and *bmp7* were also upregulated. At day 21 after injury, only five genes were significantly regulated in *CCL3*^*−/−*^ mice, again all of them reduced. Only one factor was regulated at both time points, which is thrombopoietin. While it was unexpected to detect the regulation of a protein involved in megakaryocyte maturation, thrombopoietin has previously been shown to be elevated in systemic lupus erythematosus and where it was strongly correlated with and likely induced by CCL3 [63].

Overall, at a time point when *CCL3*^*−/−*^ mice and wild-type were behaviorally distinct, we detected a strong reduction in various cytokines and chemokines, the majority of which were pro-inflammatory and, in some cases, known to have negative impact after SCI. After day 14 post-SCI, when behavioral differences were reduced, most of the differentially regulated inflammatory factors also disappeared. It is possible that the abolishment of the early, high peak of CCL3 expression had the strongest impact in reducing cellular infiltration and progressive tissue damage, which is reflected in reduced lesion size and neuronal damage as well as transient functional improvement. While an inflammatory reaction is important for tissue homeostasis and recovery, the prolonged, unresolved, and exacerbated inflammation in spinal cord contusion injuries is related to pro-inflammatory responses, extensive secondary damage, and a negative impact on functional recovery [73, 96].

Interestingly, the differences in inflammatory cytokines observed at earlier time points are no longer detectable after day 10, which is followed by the loss of the behavioral difference detected shortly afterwards. This could be indicative of a CCL3 mediated, indirect effect via other pro-inflammatory cytokines. The altered inflammatory environment in the injured spinal cord likely contributes to reduced secondary tissue damage after SCI. However, reduction of CCL3 levels at later time points might be insufficient to maintain robust behavioral differences. A reason for this might also lie in the limitations of the model. It cannot be excluded that, while axons are preserved in the absence of CCL3, these are not sufficient or required for functional recovery. It is possible that the preservation of additional pathways would be necessary in order to obtain a robust functional improvement.

To our knowledge, this is the first study using *CCL3*^*−/−*^ mice to investigate the impact of CCL3 on inflammation, secondary tissue damage and functional recovery after SCI. Taken together, the CCL3 pathway is an interesting target to reduce inflammation and decrease secondary damage after SCI.

## Conclusion

The chemokine CCL3 is involved in the inflammatory response in traumatic, inflammatory, and degenerative conditions of the CNS. After SCI, prolonged and exacerbating inflammation contributes to the secondary tissue damage. In this study, we show that the absence of CCL3 results in a mild but significant functional improvement after SCI together with smaller lesion size that was sustained over time followed by a markedly reduced pro-inflammatory response. Our findings highlight the contribution of this pro-inflammatory chemokine in maintaining the exacerbated immune response after SCI.

## Supplementary Information

**Additional file 1: Supplemental Figure 1**. Dynamic changes in gene expression of CCL3 and its receptors following SCI. After milder contusion SCI (40 kdyne), CCL3 was significantly upregulated starting 1hr and up to 28d following injury compared to uninjured (laminectomy only) mice, except for day 3 and 7. CCR1 was also upregulated beginning at 1hr and up to 3d post SCI. CCR4 was only upregulated at 6hr after injury, and CCR5 was upregulated starting at 1hr and stayed significantly upregulated until day 28. Results were analyzed using the ΔΔct method by normalizing results to a housekeeping gene (PPIA) and expressed as fold changes compared to laminectomy control. Data are expressed as mean ± SEM. * < *p*-value 0.05, one-way ANOVA, *n*= 5-6 animals/group.

**Additional file 2: Supplemental Figure 2**. No changes in gene expression of CCL3 and its s at different timepoints after laminectomy. Neither CCL3 nor its receptors showed any significant gene expression change starting at 6 hours and up to 7 days post laminectomy compared to spinal cords from naive mice without any surgical procedure. Results were analyzed using the ΔΔct method by normalizing results to a housekeeping gene (PPIA) and expressed as fold changes to naïve negative control. Data are expressed as mean ± SEM. * *p*-value< 0.05 by one-way repeated measures ANOVA followed by Tukey’s or Sidak’s method for comparison between groups, *n*= 5 animals/ group.

## Data Availability

All data supporting the conclusion of the article are included in this article.

## Abbreviations

BMS: Basso mouse scale
Bmp: Bone morphogenetic protein
CCL3: Chemokine ligand 3
CD: Cluster of differentiation
CSPG: Chondroitin sulfate proteoglycan
EAE: Experimental autoimmune encephalomyelitis
HCl: Hydrogen chloride
IL: Interleukin
LPS: Lipopolysaccharide
MIP-1α: Macrophage inflammatory protein 1- α
Mif: Macrophage migration inhibitory factor
MS: Multiple sclerosis
PFA: Paraformaldehyde
SCI: Spinal cord injury
TBS-T: Tris-buffered saline and Tween
TNF: Tumor necrosis factor
Q-PCR: Quantitative real-time PCR
