# Correction to: CCL3 contributes to secondary damage after spinal cord injury

**DOI:** 10.1186/s12974-021-02128-9

**Published:** 2021-04-03

**Authors:** Nicolas Pelisch, Jose Rosas Almanza, Kyle E. Stehlik, Brandy V. Aperi, Antje Kroner

**Affiliations:** 1 Department of Neurosurgery, Medical College of Wisconsin, Milwaukee, WI 53226 USA; 2 Clement J. Zablocki Veterans Affairs Medical Center, Milwaukee, WI 53295 USA; 3 Department of Microbiology and Immunology, MedicalCollege of Wisconsin, Milwaukee, WI 53226 USA

**Correction to: J Neuroinflammation 17, 362 (2020)**

**https://doi.org/10.1186/s12974-020-02037-3**

Following publication of the original article [1], the authors noticed that there was a covered up area which seems to be hiding something found in Fig. 7a. The original article has been corrected.

**Fig. 7:**
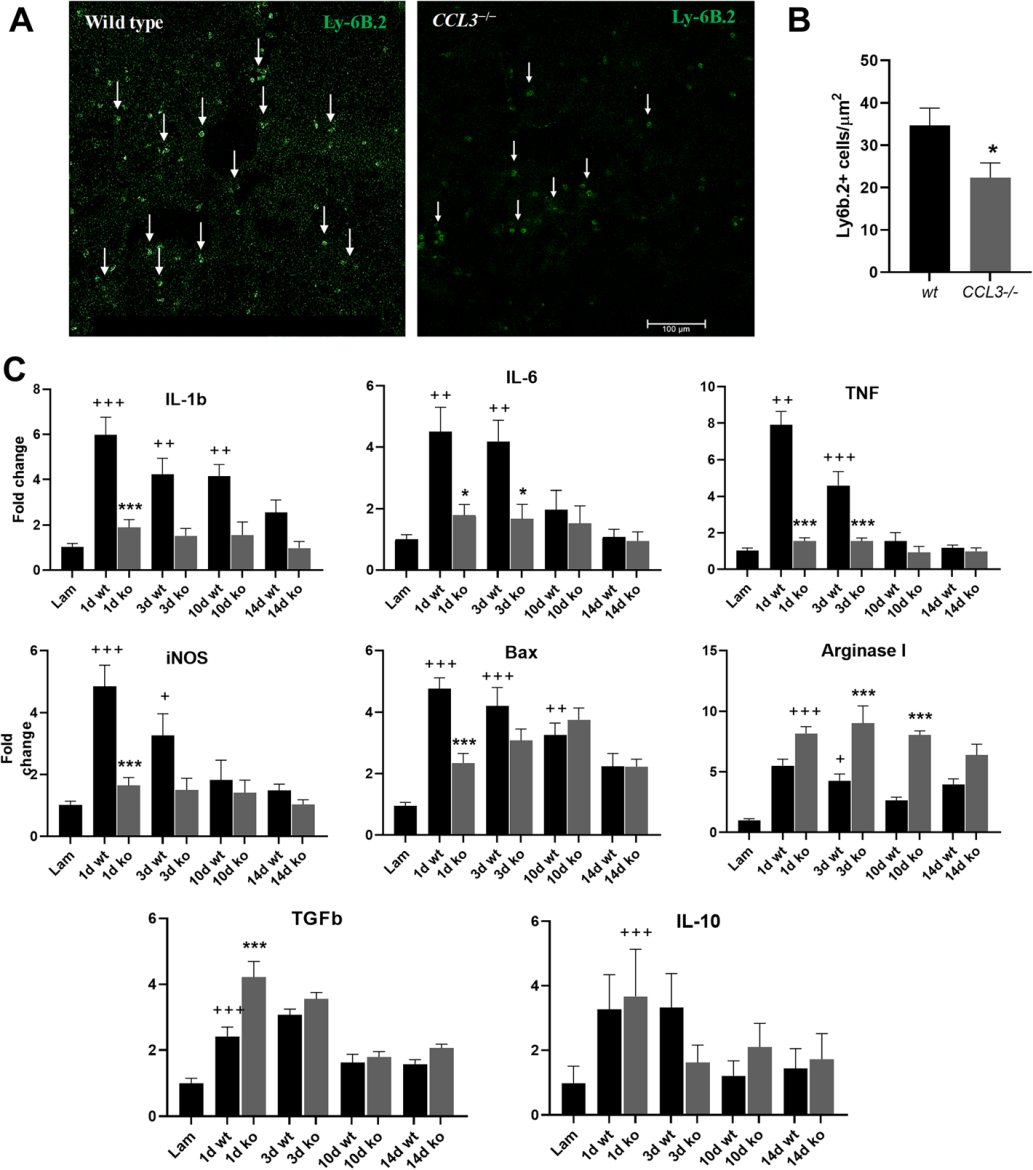
Inflammatory response in *CCL3*^−/−^ mice is reduced after SCI. **a** Representative images of Ly-6B.2-positive neutrophils in wild-type and *CCL3*^*–/–*^ SCI tissue at the lesion epicenter at day 1 after injury. Scale bar = 100 μm. Arrows indicate Ly-6B.2-positive profiles. **b** Quantitative analysis of neutrophil recruitment shows a significant reduction of neutrophils at the lesion epicenter of *CCL3*^*–/–*^ mice. *n* = 3/group. * = *p* value < 0.05. **c** Expression levels of the pro-inflammatory cytokines *il-1b*, *il-6*, *tnf*, *inos*, and the apoptotic marker *bax* were significantly increased at different time points in wild-type mice compared to laminectomy controls. *CCL3*^*–/–*^ mice, however, showed significantly lower expression levels compared to wild-type. *Arginase-1* and *TGFb*, which can indicate anti-inflammatory properties, were significantly upregulated in *CCL3*^*–/–*^ mice in relation to wild-type mice. IL-10 was upregulated compared to the laminectomy control but did not differ between genotypes. *n* = 6 wild-type, 5 *CCL3*^*–/–*^ mice. + = *p* value < 0.05, ++ = *p* value < 0.01, +++ = *p* value < 0.001 (wild-type compared to laminectomy control), * < *p* value 0.05, *** < *p* value 0.0001 (*CCL3*^*–/–*^ compared to wild-type, same day)
