# Supplementary figures and images for: CCL3 contributes to secondary damage after spinal cord injury
Source: J Neuroinflammation. 2020 Nov 27;17:362. doi: 10.1186/s12974-020-02037-3 (PMC7694914; doi:10.1186/s12974-020-02037-3)

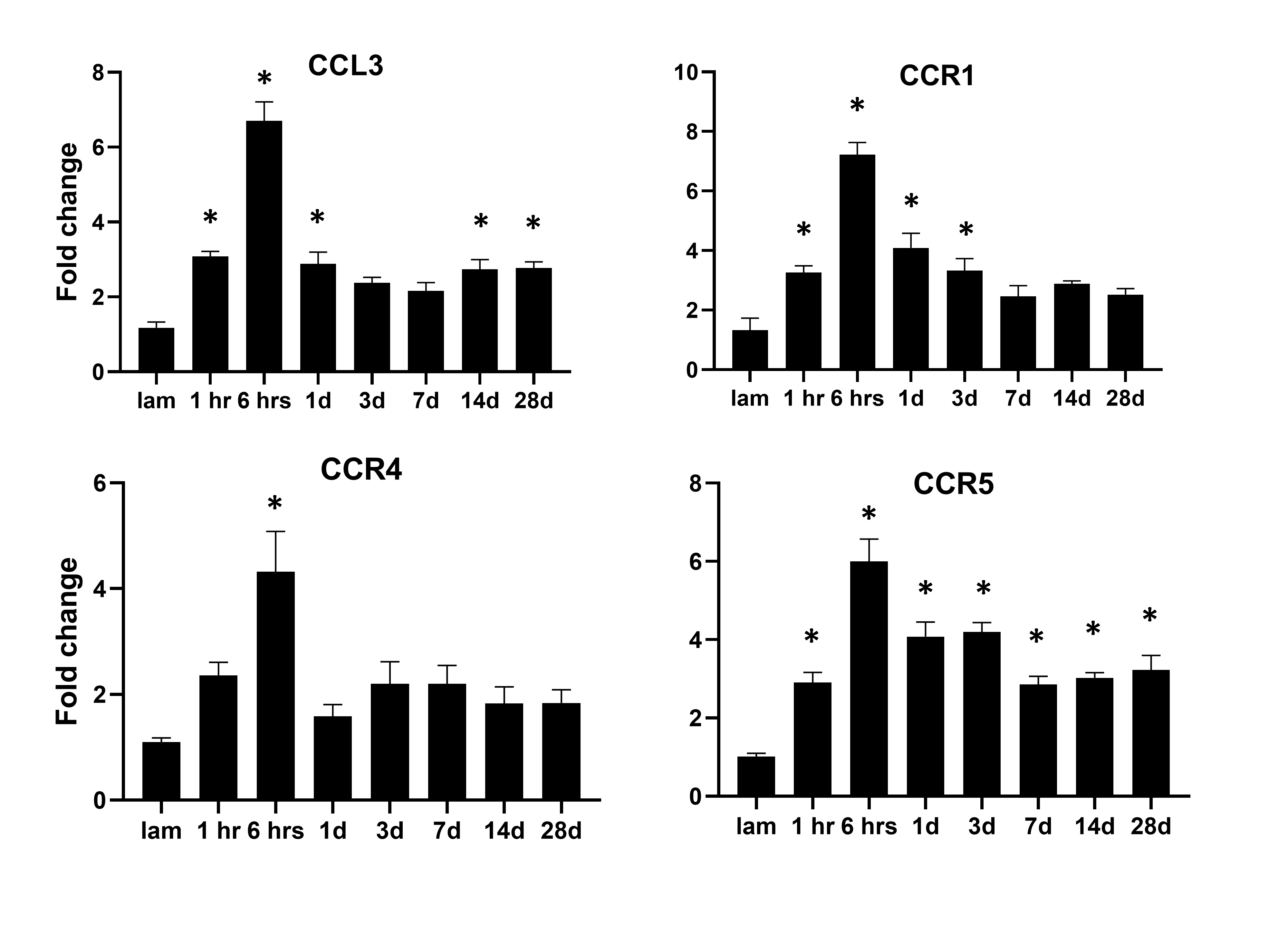

Supplement: Supplementary file 1 — Additional file 1: Supplemental Figure 1. Dynamic changes in gene expression of CCL3 and its receptors following SCI. After milder contusion SCI (40 kdyne), CCL3 was significantly upregulated starting 1hr and up to 28d following injury compared to uninjured (laminectomy only) mice, except for day 3 and 7. CCR1 was also upregulated beginning at 1hr and up to 3d post SCI. CCR4 was only upregulated at 6hr after injury, and CCR5 was upregulated starting at 1hr and stayed significantly upregulated until day 28. Results were analyzed using the ΔΔct method by normalizing results to a housekeeping gene (PPIA) and expressed as fold changes compared to laminectomy control. Data are expressed as mean ± SEM. * < p-value 0.05, one-way ANOVA, n= 5-6 animals/group. [file 12974_2020_2037_MOESM1_ESM.jpg]

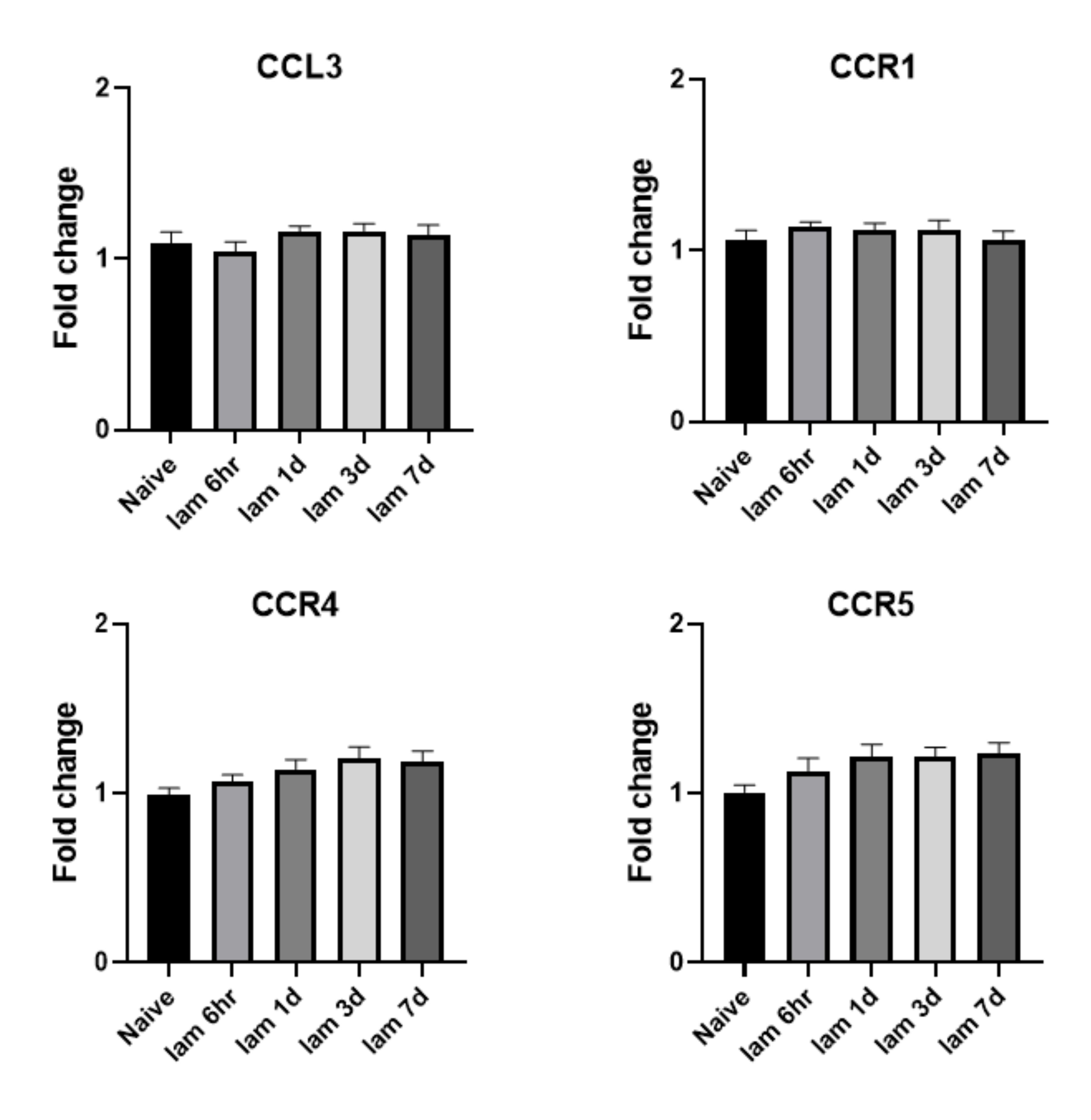

Supplement: Supplementary file 2 — Additional file 2: Supplemental Figure 2. No changes in gene expression of CCL3 and its s at different timepoints after laminectomy. Neither CCL3 nor its receptors showed any significant gene expression change starting at 6 hours and up to 7 days post laminectomy compared to spinal cords from naive mice without any surgical procedure. Results were analyzed using the ΔΔct method by normalizing results to a housekeeping gene (PPIA) and expressed as fold changes to naïve negative control. Data are expressed as mean ± SEM. * p-value< 0.05 by one-way repeated measures ANOVA followed by Tukey’s or Sidak’s method for comparison between groups, n= 5 animals/ group. [file 12974_2020_2037_MOESM2_ESM.jpg]
